# Effect of prophylactic non-invasive mechanical ventilation on functional capacity after heart valve replacement: a clinical trial

**DOI:** 10.6061/clinics/2017(10)05

**Published:** 2017-10

**Authors:** Amaro Afrânio de Araújo-Filho, Manoel Luiz de Cerqueira-Neto, Lucas de Assis Pereira Cacau, Géssica Uruga Oliveira, Telma Cristina Fontes Cerqueira, Valter Joviniano de Santana-Filho

**Affiliations:** I Nucleo de Pos Graduacao em Ciencias da Saude, Universidade Federal de Sergipe, Aracaju, SE, BR; II Departamento de Fisioterapia, Universidade Tiradentes - UNIT, Aracaju, SE, BR; III Departamento de Fisioterapia, Universidade Federal de Sergipe - UFS, Aracaju, SE, BR; IV Departamento de Fisioterapia, Universidade Federal de Sergipe - UFS, Lagarto, SE, BR

**Keywords:** Thoracic Surgery, Continuous Positive Airway Pressure, Walk Test

## Abstract

**OBJECTIVE:**

During cardiac surgery, several factors contribute to the development of postoperative pulmonary complications. Non-invasive ventilation is a promising therapeutic tool for improving the functionality of this type of patient. The aim of this study is to evaluate the functional capacity and length of stay of patients in a nosocomial intensive care unit who underwent prophylactic non-invasive ventilation after heart valve replacement.

**METHOD:**

The study was a controlled clinical trial, comprising 50 individuals of both sexes who were allocated by randomization into two groups with 25 patients in each group: the control group and experimental group. After surgery, the patients were transferred to the intensive care unit and then participated in standard physical therapy, which was provided to the experimental group after 3 applications of non-invasive ventilation within the first 26 hours after extubation. For non-invasive ventilation, the positive pressure was 10 cm H_2_O, with a duration of 1 hour. The evaluation was performed on the 7^th^ postoperative day/discharge and included a 6-minute walk test. The intensive care unit and hospitalization times were monitored in both groups. Brazilian Registry of Clinical Trials (REBeC): RBR number 8bxdd3.

**RESULTS:**

Analysis of the 6-minute walk test showed that the control group walked an average distance of 264.34±76 meters and the experimental group walked an average distance of 334.07±71 meters (*p*=0.002). The intensive care unit and hospitalization times did not differ between the groups.

**CONCLUSION:**

Non-invasive ventilation as a therapeutic resource was effective toward improving functionality; however, non-invasive ventilation did not influence the intensive care unit or hospitalization times of the studied cardiac patients.

## INTRODUCTION

Cardiac surgeries can cause a series of clinical and functional complications that are linked to specific factors, such as general anesthesia, median sternotomy, cardiopulmonary bypass and thoracic manipulation. Postoperative pulmonary complications are the most common complication type and, in turn, contribute directly to increased morbidity and mortality and longer hospital stays 1-3. These dysfunctions alter breathing patterns, which reduces the lung volume and capacity, and contribute to the appearance of atelectasis and to changes in the ventilation/perfusion ratio, which reduces the cardiorespiratory capacity and promotes physical inactivity, loss of muscle strength and loss of physical conditioning 4,5. Thus, cardiac rehabilitation plays an important role in the safe and effective treatment of these patients, reduces mortality, hospitalizations and hospital costs and improves symptoms and quality of life. A cardiac rehabilitation program should focus on improving the physiological state, functional ability and psychology of the patient and be based on a multidisciplinary approach 3-6.

As an important component of this multidisciplinary approach, physical therapy is used to both prevent and treat complications by employing techniques and therapeutic procedures to restore a functional respiratory pattern and the physical independence of the patient, either in the hospital bed or on an ambulatory basis 4,7,8. Among the therapeutic resources used, non-invasive ventilation (NIV) has shown satisfactory results. NIV has the main objective of providing ventilatory assistance to improve oxygenation and/or decrease CO_2_ retention, which reduces breathing efforts, anaerobic metabolism and the dyspnea index, avoiding endotracheal intubation and increasing residual volume and alveolar recruitment, thus preventing the presence of atelectasis and maintaining adequate gas exchange 4,9-12. NIV can increase physical performance in such patients by increasing oxygenation in the microcirculation of peripheral muscles, which reflects a better contractile performance of the heart muscle and subsequently provides better perfusion of peripheral tissues, including muscles; thus, exercise tolerance is increased 9,13-17.

Given the aforementioned data describing the use of NIV during the cardiac postoperative period, information regarding the mechanisms of action of NIV in the functional capacity of these patients is lacking, and whether there are advantages to NIV use as a preventive therapy during the postoperative period is unknown. Thus, this study aimed to evaluate the functional capacity and length of stay of patients in the nosocomial intensive care unit (ICU) and the lengths of hospital stays of patients after heart valve replacement who had undergone three prophylactic one-hour applications of NIV in CPAP mode with a pressure level of 10 cm H_2_O. We hypothesized that this therapeutic resource would improve the functional performance of these patients.

## MATERIALS AND METHODS

### Research Setting

The study was conducted at the Cardiovascular ICU and Cardiology Office of Charitable Foundation of Surgery Hospital (Fundação de Beneficência Hospital de Cirurgia – FBHC) located in Aracaju-SE, which participates in the Brazilian Unified Health System (Sistema Único de Saúde – SUS) as a referral for the care of cardiovascular surgery in the state of Sergipe and in the cities of the states of Bahia and Alagoas.

### Study Design

The study was a randomized clinical trial. A quantitative approach was used to assess the use of non-invasive mechanical ventilation as a prophylactic therapeutic resource for the functional capacity of patients in a cardiac postoperative period.

**Figure 1:**
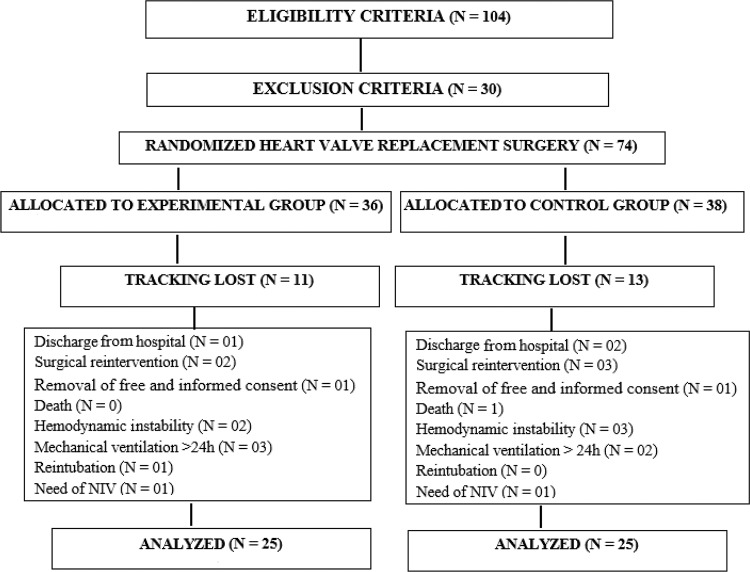
Algorithm for patient distribution (CONSORT). Source: research data.

The sample consisted of adult patients of both sexes who were randomized into two groups (an experimental group and a control group, with 25 patients in each group) through an electronic system (http://randomizer.org/form.htm) as the patients underwent hospital admission to undergo heart valve replacement surgery. To guarantee concealment of the allocation list, the randomization was performed by an independent researcher (Figure 1).

### Blinding

Considering the type of our intervention, it was not possible to blind the patients and the investigators that performed the prophylactic CPAP. However, the investigators that assessed the outcomes were blinded.

### Outcomes

The primary outcome of this study was the maximum walked distance in the six-minute walking test (6MWT), and the secondary outcome was the length of the hospital stay. The walking test was assessed at hospital discharge. The length of stay was assessed at intensive care discharge and at hospital discharge.

### Population

This study was carried out in a reference hospital for cardiac surgery of the Brazilian Public Health System (Sistema Único de Saúde - SUS, Fundação de Beneficência Hospital de Cirurgia - FBHC, Aracaju, Sergipe).

The eligibility criteria were as follows: patients were admitted to the hospital for heart valve replacement surgery; comprised both genders; and were between 20 and 70 years old.

The exclusion criteria were as follows: patients with motor disabilities that prevented walking and patients with contraindications to CPAP, such as a decreased level of consciousness, an ineffective cough, an airway obstruction, abdominal distention, vomiting, upper gastrointestinal bleeding, acute coronary syndrome, complex arrhythmias, facial trauma, esophageal surgery and an undrained barotrauma.

Sources of lost tracking (discontinuity) included the following: early discharge from the hospital; need for reintubation; need for invasive mechanical ventilation more than 24 hours after surgery; non-invasive need for ventilation therapeutically for reversal of respiratory failure; surgical complications or reintervention; hemodynamic instability; death; and removal of free and informed consent. At any time in the study, patients from the control group who showed a need for invasive or non-invasive ventilatory support for dyspnea (i.e., increased respiratory rate >25 bpm, paradoxical pattern, use of accessory muscles and decrease in peripheral saturation oxygen) were removed from the study.

Based on the eligibility criteria, 104 patients were included in this study, and 30 patients were removed after the exclusion criteria analysis. A total of 74 patients were randomized; 36 were allocated to the experimental group, and 38 were allocated to the control group. Of these patients, 11 from the experimental group and 13 from the control group were affected by the research discontinuation criteria; 50 patients remained for the final analysis of the present study and were distributed as follows: 25 in the experimental group, and 25 in the control group (Figure 1).

### Sampling Technique

Patients were randomized into two groups (the experimental group and the control group) using a randomization process produced by an electronic system: http://randomizer.org/form.htm. At the time of hospitalization, each patient underwent a preoperative assessment and an assessment protocol that consisted of patient identification, vital signs, clinical diagnosis, surgery type, medical history, medications and the preoperative ejection fraction (EF).

Postoperative revaluation was performed on the 7^th^ postoperative day (POD) or at discharge. The 6MWT was performed, and the operative times, extracorporeal circulation (ECC), ICU and hospitalization times, as well as the postoperative EFs of both groups, were assessed.

After surgery, the patients were transferred to the ICU, where they were subjected to the interruption process of mechanical ventilation. The patients in the experimental group received three applications of NIV within the first 26 hours after extubation, and the first application was performed within the first 2 hours after the removal of the tube, maintaining an interval of 12 hours between the other applications. For therapy, a positive pressure device, ResMed S7 Lightweight in Continuous Positive pressure mode (CPAP), and an oronasal interface were used for one hour and adjusted with a PEEP of 10 cm of H_2_O 9,18. In addition to the NIV application, patients in the experimental group underwent the physical therapy protocol, which was performed by the hospital's physiotherapy team based on the guidelines of the Brazilian Society of Cardiology, throughout their hospitalization. The control group did not undergo NIV therapy; these patients participated in respiratory and motor therapy only.

### Instrument

#### The 6MWT

The 6MWT is a simple test that is easy to perform, safe, inexpensive and consists of a patient walking 30 meters for 6 minutes in a corridor. To assess his or her tolerance to physical exertion, the test was performed with a brisk walk, but no running, and the patient was verbally encouraged during each minute of the test to complete the maximum distance. The Borg Scale was used before and after the 6 minutes to verify the patient's subjective fatigue 19.

To prepare for the test, checks were performed at zero, 3 and 6 minutes. At zero minutes, vital signs such as blood pressure (BP), heart rate (HR), respiratory rate (RR) and oxygen saturation (SpO_2_) were measured. At the third minute, only SpO_2_ and HR were measured because they needed to be checked while walking, without affecting the patient's performance. At no time did the evaluator interrupt the patient, which was possible with use of a pulse oximeter. At the sixth minute, all variables were verified (BP, RR, HR and SpO2), and the patient's fatigue level was evaluated immediately after termination 20,21.

### Ethical Aspects

Patients only participated in the study after providing either signed or fingerprint-marked informed consent directly or by proxy through a guardian (family and/or companion). The informed consent form consisted of a declaration informing the subject of the research, objectives, evaluation methods and procedures, risks and benefits.

Each patient received an explanation regarding how the research could contribute positively to his or her recovery process and to clarify possible doubts. The patients were informed that they could withdraw from research participation whenever they wanted. The patients were also warned that, at the time of the NIV application, respiratory distress and fatigue may occur.

The study was approved by the Ethics Committee on Human Research of Tiradentes University in accordance with the Declaration of Helsinki under Protocol number 021211R. The study was published in the Brazilian Registry of Clinical Trials (REBeC) with the RBR number 8bxdd3.

### Statistical Analysis

Data were categorized in Microsoft Excel 2007® spreadsheet files, and for the statistical analysis, BioEstat 5.0 was used. The normality condition was assessed using the Shapiro-Wilk test. Student’s t-test was used to compare the parametric data, and the Mann-Whitney test was used for nonparametric samples. For comparisons of categorical variables, such as sex, a chi-squared test was used, and *p* values <0.05 were considered statistically significant.

The study by Oliveira et al. 3 was used as the basis for the calculation of sample size. The distance walked in the 6MWT was considered the main parameter. Assuming that the effect size=50 meters, variability=50.66 meters, significance level=5%, and power test=95%, a minimum sample size of 19 patients for each group was determined.

## RESULTS

Table 1 shows the data for the characterization of the sample relating to the experimental group and control group, considering age, gender, pre-surgical EF of the left ventricle (LVEF) and ECC time. The results demonstrated that there was no statistically significant difference between the groups, even with the homogeneity of the patients included in the study. Table 1 shows the length of stay in the ICU and total hospital stay times of the experimental and control groups. Regarding the ICU times, the length of stay for the experimental group length was 3 days 2, 3, and the length of stay for the control group was 3 days 2, 3 (*p*=0.41). In relation to hospitalization times, the length of stay for the experimental group was 7 days 7, 9, and the length of stay for the control group was 8 days 6, 5, 8 (*p*=0.55). These data showed that there was no statistically significant difference between the groups.

**Figure 2:**
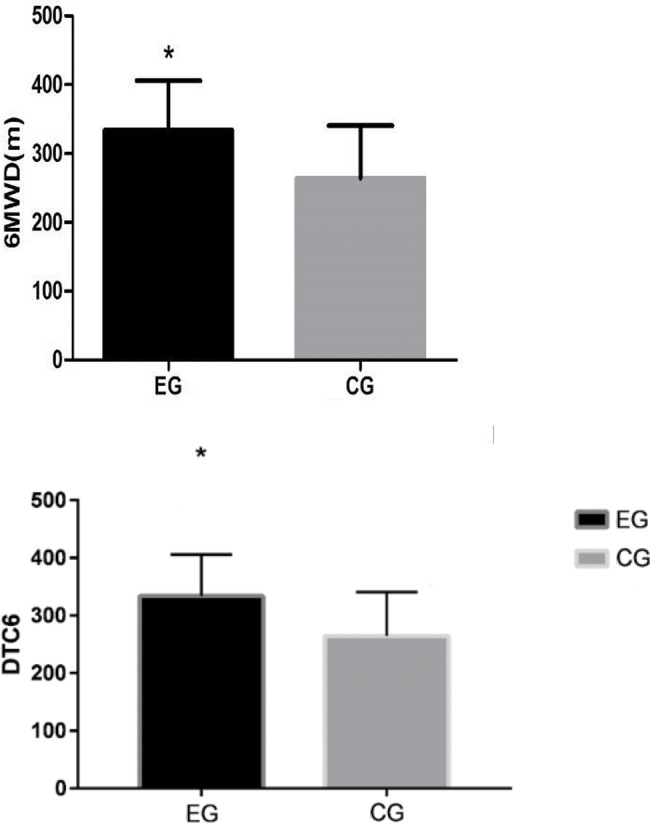
Comparison of the distance covered IN the 6MWT by the experimental group (EG) and control group (CG). A *t*-test was performed for the independent samples (*p<0.05).

Figure 2 shows the 6MWT results. The experimental group (334.07±71 meters) covered a significantly greater distance than the control group (264.34±76 meters).

## DISCUSSION

In this study, we evaluated the functional capacities, lengths of stay in the ICU and lengths of stay in the hospital of the patients after heart valve replacement and three applications of prophylactic NIV within the first 26 hours after extubation. The patients in the experimental group walked 334.07 meters in the 6MWT, whereas the patients from the control group walked 264.34 meters, which represented a significant difference. As a secondary outcome, no difference was detected between the groups for length of stay in the hospital or in the ICU. These findings demonstrate that the use of CPAP in the immediate postoperative period in patients subjected to heart valve replacement improves functional activity, as evidenced by a longer distance walked in the 6MWT at hospital discharge.

NIV has been extensively used in early-stage acute respiratory failure (ARF), and the success of treatment has been associated with the ARF type and severity, underlying disease, application time, treatment site, and the experience of the team 14,15,18,22-25. However, reports describing the prophylactic use of NIV are lacking.

The study by Zarbock et al. 10 demonstrated that administration of NIV for an extended period of time after cardiac surgery improved arterial oxygenation, reduced the incidence of pulmonary complications, including pneumonia and the need for reintubation, and reduced the rate of readmission to the ICU, which may explain the superior performance in the walking test of the patients who underwent NIV in our study.

Using a similar methodological design and population, Mazullo Filho et al. 26 carried out a randomized, controlled study with 32 patients during the immediate postoperative period after cardiac surgery. The experimental group used NIV for 2 hours after extubation. The authors found an average reduction in HR and a lower cardiac output, thus reducing the need for energy expenditure and respiratory effort, compared to the control group, which did not use NIV. With lower energy expenditure and lower RR than patients without NIV, the cardiopathy patient with NIV can exhibit better cardiopulmonary fitness and perform his or her activities in a more functional way, which is the primary outcome of our research.

Functional capacity has been evaluated in research studies of patients with heart failure. The 6MWT is a useful tool for this purpose because the test is simple and cost-effective. The 6MWT can be used to reproduce daily activities, evaluate exercise tolerance, assess the degree of functional limitations, and enable prognostic stratification 27,28. Thus, the 6MWT was chosen as an evaluation tool in this study. Additionally, the 6MWT is sensitive for the detection of better exercise tolerance in patients who are subjected to CPAP during the postoperative stage of heart valve replacement.

A meta-analysis by Bundchen et al. 16 revealed only three studies that used NIV after heart failure surgery. The primary outcome was the distance traveled during the 6MWT, and all of the studies showed positive results. Wittmer et al. 29 compared a program of breathing exercises associated with 30 minutes of CPAP (8 cm H_2_O) *versus* only breathing exercises for a period of 14 days. Chermont 30 compared 30 minutes of CPAP (4-6 cm H_2_O) *versus* placebo before the 6MWT, and Lima et al. 31 showed that the use of CPAP (10 cm H_2_O) for 30 minutes before the 6MWT increased the distance traveled compared to that of the control group without the use of CPAP. With the same primary outcome, our study used a different methodological design, comprising three one-hour CPAP applications (10 cm H_2_O) in the patient who was still hospitalized in the ICU, and obtained positive results, evidenced by an increase in the distance walked during the 6MWT carried out at discharge. This reinforces the idea of making CPAP a standard procedure in the immediate postoperative period of cardiac surgery.

Functional capacity is limited in patients with cardiac disease, not only due to cardiac factors but also due to the interaction between the cardiopulmonary and musculoskeletal systems. Abnormal ventilatory responses are common in these patients, and combined with peripheral muscle dysfunction, these responses result in greater difficulty in performing the activities of daily life, especially in the cardiac postoperative period, which is the focus of our research. NIV can reduce respiratory effort and increase physical performance in such patients by increasing oxygenation in the microcirculation of peripheral muscles that can lead to increased intrathoracic pressure, reducing the transmural pressure of the left ventricle and consequently the pre- and post-load pressure. This process reflects better contractile performance of the heart muscle, providing better perfusion of peripheral tissues, including muscles, and thus causes an increase in exercise tolerance 9,14-17. This process has been demonstrated as our primary outcome when patients who received prophylactic CPAP walked longer distances than patients who did not receive prophylactic CPAP.

According to Morales-Blanhir et al. 32, through a systematic review of the 6MWT, the average distance traveled by healthy people is 550 meters. Oliveira et al. 3 affirms that patients who perform the 6MWT during the cardiac postoperative stage have an average distance of 375 meters. A literature review by Zielinska et al. 33 shows that the 6MWT for cardiac patients is a reliable measure and a predictor of mortality, demonstrating that patients who travel a distance of fewer than 350 meters have a higher mortality rate. In a retrospective study using data from 330 surgical cardiac patients, with 97 of them undergoing heart valve replacement, La Rovere et al. 34 analyzed pre- and post-surgery 6MWT results and obtained values of 256 meters and 381 meters before and after heart valve replacement surgery, respectively. Our research showed that patients in the NIV group traveled 334.04 meters, while patients in the control group reached 264.34 meters. Our results are within the reference values of the literature; however, these values are very different from the values for healthy individuals, demonstrating the great functional loss of cardiac patients, highlighting the need to maintain cardiac rehabilitation (phase two) of these patients after hospital discharge.

When evaluating the secondary outcomes of our study, such as length of stay in the ICU and the total hospital stay of patients during the postoperative period after heart valve replacement surgery, no significant differences in these times were evident between the groups. Trevisan et al. 35, in a randomized clinical trial, and Landoni et al. 9 and Cabrini et al. 24, in systematic reviews, showed that the use of NIV when applied post-extubation does not interfere with the length of stay in the ICU or in the hospital. In our study, we found a slight, but not significant, reduction in the number of days. These data may be explained by the profiles of the studied patients, who had low EFs, hypoactivity, and important muscle strength deficits that were linked to heart failure, which are factors that the therapeutic tool cannot fully revert.

This study was limited by the absence of a preoperative evaluation of the distance covered in a 6MWT because the patients were in a serious health state, debilitated and with preoperative restrictions. Another limitation was that the average time for application of CPAP was 60 minutes. This timeframe suggested the use of BPAP to promote greater ventilation comfort and thus apply NIV for a longer time and perhaps under higher pressure. Another limitation was the absence of variables to evaluate lung function so that we could correlate the improvement in functionality with the likely improvement in lung volume and capacity. Thus, we suggest further research to answer these questions and develop new therapeutic approaches in cardiac rehabilitation.

As a prophylactic therapeutic approach, NIV has been confirmed to be effective in improving the functional capacity of patients during the postoperative period of heart valve replacement surgery, as measured by the distance covered in a 6MWT. However, NIV as a therapeutic resource did not affect the length of stay in the ICU or the length of the total hospital stay of the studied cardiac patients.

## AUTHOR CONTRIBUTIONS

De Araújo-Filho AA conceived, designed, and executed the study and prepared the manuscript. De Cerqueira-Neto ML, Cacau LA, Cerqueira TC assisted in the design and critical review of the study. De Santana-Filho VJ supervised the study, conceived, designed and critically reviewed the manuscript. Oliveira GU participated in the design, analysis and interpretation of the data collected.

## Figures and Tables

**Table 1:** Study participant characteristics.

	NIV	Control	*p*
Age	42.44 ± 11	46.08 ± 14.23	0.31
Gender			
Male n (%)	16 (64.0)	11 (44.0)	0.42
Female n (%)	9 (36.0)	14 (56.0)	0.41
ICU* time (days)	3 [2, 3]	3 [2, 3]	0.49
Hospitalization time (days)	7 [7, 9]	8 [6, 5, 8]	0.12
Preoperative LVEF** (%)	56.84 ± 11.0	57.36 ± 12.1	0.87
ECC*** time (min.)	92.6 ± 41.9	81.65 ± 26.8	0.51

*ICU: Intensive care unit; **LVEF: left ventricular ejection fraction; ***ECC: extracorporeal circulation. Held Test *t* for parametric variables (age and LVEF) and Mann-Whitney test for nonparametric ones (ICU time, hospitalization time and ECC time) and Chi-squared test for the categorical variable (gender) (*p<0.05).
